# A second dose of a measles-mumps-rubella vaccine administered to healthy four-to-six-year-old children: a phase III, observer-blind, randomized, safety and immunogenicity study comparing GSK MMR and MMR II with and without DTaP-IPV and varicella vaccines co-administration

**DOI:** 10.1080/21645515.2018.1554971

**Published:** 2019-02-20

**Authors:** The MMR-158 Study Group

**Affiliations:** See Contributors section

**Keywords:** Measles-mumps-rubella vaccine, DTaP-IPV, varicella vaccine, immunogenicity, safety, co-administration

## Abstract

In many countries, a second dose of a combined measles, mumps, and rubella (MMR) vaccine is recommended at 4–6 years of age – similarly to the booster of diphtheria, tetanus, acellular pertussis, and inactivated polio vaccine (DTaP-IPV) and the second dose of varicella vaccine (VV). Vaccine co-administration is generally encouraged if no interferences exist among the vaccines. This phase IIIa, randomized, controlled trial (NCT01621802) evaluated the immunogenicity and safety of MMR-RIT (*Priorix*, GSK) when given as a second dose with or without co-administration of DTaP-IPV and VV, using MMR II (*M-M-R II*, Merck & Co Inc.) as comparator. Antibody geometric mean concentrations or titers (GMCs/GMTs) and response rates to the components of all the administered vaccines were assessed. Solicited, unsolicited, and serious adverse events were recorded. Four thousand eleven children aged 4–6 years were enrolled. MMR-RIT elicited immune responses that were not inferior to those of MMR II in terms of GMCs and seroresponse rates when administered alone or when co-administered with DTaP-IPV and VV. The immune responses to the co-administered vaccines in MMR-RIT recipients were non-inferior to those in MMR II recipients. MMR-RIT and MMR II demonstrated similar reactogenicity profiles; the most frequent solicited adverse events across vaccine groups and sub-cohorts were local pain and fever. In conclusion, the immunogenicity and safety profiles of MMR-RIT administered with or without DTaP-IPV and VV were similar to those of MMR II.

## Introduction

To prevent the highly contagious measles, mumps, and rubella diseases, the World Health Organization (WHO) and the Centers for Disease Control and Prevention (CDC) recommend maintaining a high vaccination coverage (≥80%–≥95%) with measles-, mumps-, and/or rubella-containing vaccines.^1–4^

The implementation of universal routine vaccination programs has led to a dramatic reduction in the incidence of these diseases compared to the pre-vaccine era: in the United States of America (USA), endemic measles and rubella have been eliminated and mumps cases have decreased by >99%.^5–7^ However, occasional outbreaks still occur – as observed for measles and mumps outbreaks in the recent years. Notably, mumps outbreaks were shown to occur in highly vaccinated US communities, particularly in close-contact settings.^7,8^ However, high vaccination coverage helps limit the severity, duration and spread of the disease. In this regard, MMR vaccine prevents most, but not all, cases of mumps and complications caused by the disease.

In the USA, the Advisory Committee on Immunization Practices recommends administration of a first dose of the only licensed combined measles, mumps, and rubella (MMR) vaccine, MMR II (*M-M-R II*, Merck & Co Inc.), at 12–15 months of age, followed by a second dose at 4–6 years of age.^1^ Two doses are key to maintaining a high level of overall protection. The combined measles-mumps-rubella-varicella vaccine (MMRV; *ProQuad*, Merck & Co Inc.) can also be used in the USA to protect against measles, mumps and rubella. Given the need of both high vaccination coverage and compliance with the two-dose schedule for protection, potential interruptions in MMR vaccine supply in the USA could have critical public health consequences.

In over 100 countries outside the USA, another MMR vaccine, MMR-RIT (*Priorix*, GSK), is licensed for use in individuals aged 9 months and older. A second dose of this vaccine is recommended in many countries around the time of elementary school entry.^9,10^

The recommended schedule of a second dose of MMR vaccines in many countries coincides with the timing of administration of other childhood routine vaccines such as a booster dose of diphtheria, tetanus, acellular pertussis, and inactivated polio vaccine (DTaP-IPV), and a second dose of varicella vaccine (VV). Current guidelines recommend assessing any potential interference between vaccines when administered simultaneously.^11,12^ A previous study investigated the safety of simultaneous administration of DTaP-IPV, MMR II, and VV, along with the immunogenicity of the DTaP-IPV components.^13^ However, to our knowledge, there are no published data on the immunogenicity of MMR-RIT when co-administered with both DTaP-IPV and VV.

**Figure 1.**
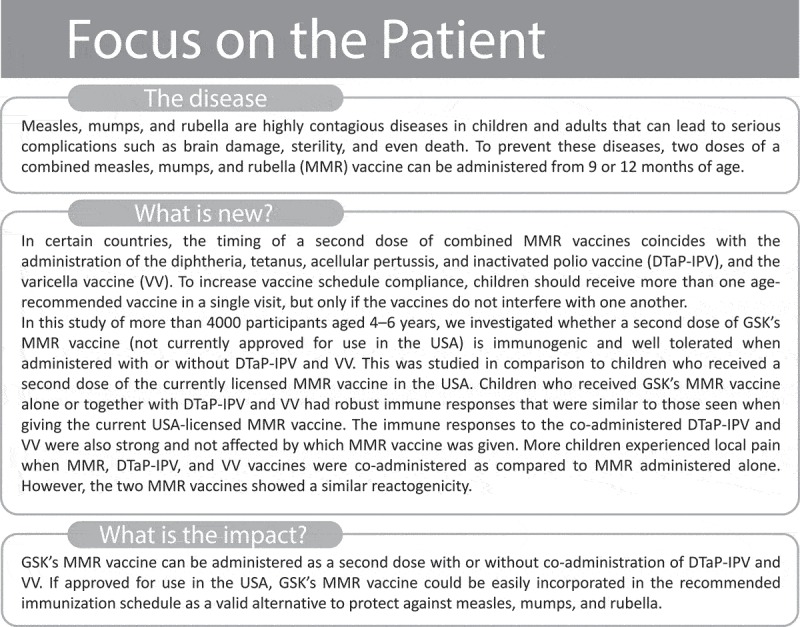
Focus on the patient section.

In this study, we evaluated the immunogenicity and safety of MMR-RIT compared to the USA standard of care, MMR II, when given as a second dose to children 4–6 years of age, with or without co-administration of DTaP-IPV (*Kinrix*, GSK) and VV (*VARIVAX*, Merck & Co Inc). A summary contextualizing the outcomes of this study is displayed in the Focus on the Patient Section (Figure 1) for the convenience of health care professionals.

## Results

### Study participants and demographic characteristics

**Figure 2.**
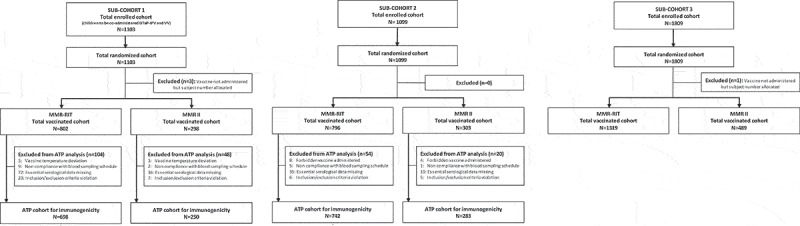
Flow diagram of the study participants in each sub-cohort. Footnote: ATP, according-to-protocol; DTaP-IPV, diphtheria, tetanus, acellular pertussis, and inactivated polio vaccine; N, number of participants; n, number of participants within the category; VV, varicella vaccine.

We enrolled a total of 4011 children in 3 different sub-cohorts to assess: immunogenicity and safety of MMR vaccine, DTaP-IPV, and VV when co-administered (sub-cohort 1); immunogenicity and safety of MMR vaccine administered alone (sub-cohort 2); and safety of MMR vaccine administered alone (sub-cohort 3) (see Patients and Methods for more details). Children in each sub-cohort were randomized and vaccinated with either MMR-RIT or MMR II in a 3:1 ratio: 1100 children in sub-cohort 1 (MMR-RIT: N = 802, MMR II: N = 298), 1099 in sub-cohort 2 (MMR-RIT: N = 796, MMR II: N = 303), and 1808 in sub-cohort 3 (MMR-RIT: N = 1319, MMR II: N = 489) (Figure 2). A total of 3846 children completed the study (1030, 1055, and 1761 in sub-cohorts 1, 2, and 3, respectively) and 161 were prematurely withdrawn (70, 44, and 47 children in sub-cohorts 1, 2, and 3, respectively). The main reasons for discontinuation were loss to follow up (MMR-RIT: ≤4.5%, MMR II: ≤5.4%) and consent withdrawal not due to an adverse event (AE) (MMR-RIT: ≤1% and MMR II: ≤1.3%). No children in this study were withdrawn due to an AE.

Within each sub-cohort, demographic characteristics were similar between the study groups (Table 1).

**Table 1.** Demographic characteristics of the study participants (total vaccinated cohort).

	Sub-cohort 1		Sub-cohort 2		Sub-cohort 3	
Characteristic	MMR-RIT (N = 802)	MMR II (N = 298)	MMR-RIT (N = 796)	MMR II (N = 303)	MMR-RIT (N = 1319)	MMR II (N = 489)
Age* (years), mean (SD)	4.1 (0.3)	4.1 (0.3)	4.4 (0.6)	4.3 (0.6)	4.4 (0.6)	4.4 (0.6)
Females:males	398:404	134:164	361:435	153:150	632:687	225:264
Country, n (%)						
South Korea	0 (0.0)	0 (0.0)	158 (19.8)	66 (21.8)	91 (6.9)	43 (8.8)
Taiwan	0 (0.0)	0 (0.0)	226 (28.4)	80 (26.4)	492 (37.3)	170 (34.8)
United States of America	802 (100)	298 (100)	412 (51.8)	157 (51.8)	736 (55.8)	276 (56.4)
Geographic ancestry, n (%)						
African heritage/African American	96 (12.0)	39 (13.1)	48 (6.0)	19 (6.3)	94 (7.1)	32 (6.5)
American Indian or Alaskan native	130 (16.2)	38 (12.8)	15 (1.9)	3 (1.0)	4 (0.3)	0 (0.0)
Asian-East Asian heritage	28 (3.5)	6 (2.0)	384 (48.2)	146 (48.2)	565 (42.8)	209 (42.7)
Asian-South East Asian heritage	49 (6.1)	25 (8.4)	11 (1.4)	4 (1.3)	16 (1.2)	8 (1.6)
White-Caucasian/European heritage	363 (45.3)	135 (45.3)	291 (36.6)	117 (38.6)	575 (43.6)	218 (44.6)
Other	136 (16.9)	55 (18.4)	47 (5.9)	14 (4.6)	65 (4.9)	22 (4.5)

N, number of participants; SD, standard deviation; n (%), number (percentage) of participants in the category.

*Age at study vaccination.

### Immunogenicity assessments

The immunogenicity of MMR-RIT and MMR II was assessed in sub-cohorts 1 and 2, while the immunogenicity of the co-administered DTaP-IPV and VV was assessed in sub-cohort 1 only. No immunogenicity assessments were performed in sub-cohort 3.

#### Non-inferiority of the immune response to MMR-RIT versus MMR II

In sub-cohort 1, MMR-RIT and MMR II were administered together with DTaP-IPV and VV. Under these circumstances, the seroresponse rates (SRRs) and antibody geometric mean concentrations (GMCs) to the MMR components at Day (D)42 were similar between vaccine groups, with SRRs of at least 99.9% for all antibodies (Table 2). MMR-RIT was non-inferior to MMR II in terms of anti-measles, anti-mumps, and anti-rubella SRRs (with seroresponse defined as an immunoglobulin G [IgG] antibody concentration ≥200 mIU/mL for anti-measles, ≥10 EU/mL for anti-mumps, and ≥10 IU/mL for anti-rubella irrespective of the baseline antibody concentrations), as the lower limits of the 97.5% confidence intervals (CIs) of the differences in SRRs (MMR-RIT minus MMR II) at D42 were above the pre-defined threshold of −5% for all 3 antibodies (Table 2).

**Table 2.** Anti-measles, anti-mumps, and anti-rubella seroresponse rates and geometric mean antibody concentrations at Day 42 when MMR-RIT and MMR II were co-administered with DTaP-IPV and VV (according-to-protocol cohort for immunogenicity, sub-cohort 1).

	SRR (%)		
	MMR-RIT (N = 697)*	MMR II (N = 249)*	Difference in SRR (MMR-RIT SRR minus MMR II SRR) % (97.5% CI)^a^
anti-measles	100	100	0.00 (−0.**72**, 1.98)
anti-mumps	100	100	0.00 (−0.**72**, 1.97)
anti-rubella	99.9	100	−0.14 (−0.**98**, 1.84)
	Adjusted GMCs		
	MMR-RIT (N = 690)*	MMR II (N = 245)*	Adjusted GMC ratio (MMR-RIT GMC over MMR II GMC) % (97.5% CI)^b^
anti-measles	4285.0	4333.5	0.99 (0.**92**, 1.06)
anti-mumps	171.3	188.5	0.91 (0.**83**, 1.00)
anti-rubella	97.1	94.5	1.03 (0.**97**, 1.09)

N, number of participants with both pre- and post-vaccination results available. * Except for anti-mumps, for which MMR-RIT (N = 698) and MMR II (N = 250) for SRR, and MMR-RIT (N = 691) and MMR II (N = 248) for GMC.

SRR, seroresponse rate: percentage of participants with antibody concentration greater than or equal to the seroresponse threshold for each assay (200 mIU/mL, 10 EU/mL, and 10 IU/mL for anti-measles, anti-mumps, and anti-rubella antibodies, respectively).

Adjusted GMC, geometric mean antibody concentration adjusted for pre-vaccination concentration.

^a^Standardized asymptotic 97.5% confidence interval.

^b^97.5% confidence interval obtained using an ANCOVA model.

Bold values indicate non-inferiority criterion met. Non-inferiority criterion for SRR: lower limit of the two-sided 97.5% CI for the group difference in SRRs at D42 (MMR-RIT SRR minus MMR II SRR) ≥-5% for measles, mumps and rubella viruses. Non-inferiority criterion for GMCs: lower limit of the two-sided 97.5% CI for the adjusted GMC ratio at D42 (MMR-RIT GMC over MMR II GMC) ≥0.67 for anti-measles, anti-mumps and anti-rubella antibodies.

In sub-cohort 1, MMR-RIT was also non-inferior to MMR II in terms of antibody GMCs to anti-measles, anti-mumps, and anti-rubella viruses, as we found that for all 3 antibodies the lower limits of the 97.5% CIs of the adjusted GMC ratios (MMR-RIT over MMR II) at D42 were above the pre-defined threshold of 0.67 (Table 2).

In sub-cohort 2, MMR-RIT and MMR II were administered alone. In this sub-cohort, SRRs and GMCs for antibodies to the MMR components were similar between vaccine groups (Table 3). All children vaccinated with MMR-RIT and ≥99.3% of children vaccinated with MMR II showed anti-measles, anti-mumps, and anti-rubella antibody seroresponses. The non-inferiority of MMR-RIT compared to MMR II was met in terms of both SRRs and GMCs for all 3 antibodies tested (the criteria used were the same as for the SRR and GMC objectives in sub-cohort 1; Table 3).

**Table 3.** Anti-measles, anti-mumps, and anti-rubella seroresponse rates and geometric mean antibody concentrations at Day 42 when MMR-RIT and MMR II were administered alone (according-to-protocol cohort for immunogenicity, sub-cohort 2).

	SRR (%)		
	MMR-RIT (N = 736)	MMR II (N = 283)	Difference in SRR (MMR-RIT SRR minus MMR II SRR) % (97.5% CI)^a^
anti-measles	100.0	99.3	0.71 (**0.02**, 2.97)
anti-mumps	100.0	100.0	0.00 (−0.**68**, 1.75)
anti-rubella	100.0	100.0	0.00 (−0.**68**, 1.75)
	Adjusted GMCs		
	MMR-RIT (N = 729)*	MMR II (N = 280)*	Adjusted GMC ratio (MMR-RIT GMC over MMR II GMC) % (97.5% CI)^b^
anti-measles	3600.3	3504.3	1.03 (**0.96**, 1.10)
anti-mumps	167.7	174.6	0.96 (**0.87**, 1.06)
anti-rubella	99.3	98.6	1.01 (**0.95**, 1.07)

N, number of participants with both pre- and post-vaccination results available. * Except for anti-mumps, for which MMR-RIT (N = 732) and MMR II (N = 282).

SRR, seroresponse rate: percentage of participants with antibody concentration greater than or equal to the seroresponse threshold for each assay (200 mIU/mL, 10 EU/mL, and 10 IU/mL for anti-measles, anti-mumps, and anti-rubella antibodies, respectively).

Adjusted GMC, geometric mean antibody concentration adjusted for country and pre-vaccination concentration.

^a^Standardized asymptotic 97.5% confidence interval.

^b^97.5% confidence interval obtained using an ANCOVA model.

Bold values indicate non-inferiority criterion met. Non-inferiority criterion for SRR: lower limit of the two-sided 97.5% CI for the group difference in SRRs at D42 (MMR-RIT SRR minus MMR II SRR) ≥-5%. Non-inferiority criterion for GMCs: lower limit of the two-sided 97.5% CI for the adjusted GMC ratio at D42 (MMR-RIT GMC over MMR II GMC) ≥0.67.

Of note, pre-vaccination antibody GMCs within each sub-cohort were comparable between the MMR-RIT and MMR II groups for anti-measles (ranging from 2729.6 to 3644.1 mIU/mL), anti-mumps (49.6–60.7 EU/mL), and anti-rubella antibodies (45.1–61.5 IU/mL).

#### Non-inferiority of MMR-RIT versus MMR II in terms of immune responses to DTaP-IPV and VV

In sub-cohort 1, DTaP-IPV and VV were co-administered with either MMR-RIT or MMR II vaccine. The doses of DTaP-IPV and VV corresponded to the fifth dose of DTaP-containing vaccine, fourth dose of IPV and second dose of VV in the recommended immunization schedule for the participants. For each MMR vaccine group in sub-cohort 1, the immunogenicity of the co-administered VV was assessed in terms of SRR and antibody GMC for anti-varicella zoster virus (VZV), and the immunogenicity of DTaP-IPV was measured in terms of booster response rates (BRRs; see definitions in Patients and Methods), GMCs, and antibody geometric mean titers (GMTs) to the antigens in the vaccine.

The immune responses to the varicella, diphtheria, tetanus, pertussis, and the 3 polio antigens at D42 were similar between the MMR-RIT and MMR II groups (Tables 4 and 5). After vaccination with MMR-RIT, ≥99.7% of children showed seroresponse to anti-VZV and ≥93.9% showed a booster response to the DTaP-IPV components (Table 4).

**Table 4.** Seroresponse rate and booster response rate to antibodies to the co-administered vaccines at Day 42 (according-to-protocol cohort for immunogenicity, sub-cohort 1).

	MMR-RIT		MMR II		Difference in BRR or SRR (MMR-RIT minus MMR II) % (97.5% CI)^a^
	N	BRR or SRR (%)	N	BRR or SRR (%)	
anti-DT	659	99.7	233	100.0	−0.30 (−1.**29**, 1.81)
anti-FHA	659	94.1	234	94.4	−0.36 (−3.**90**, 4.34)
anti-PRN	660	99.5	234	99.6	−0.03 (−1.**17**, 2.44)
anti-PT	659	97.6	233	96.6	1.01 (−1.**54**, 4.95)
anti-TT	661	93.9	234	95.7	−1.78 (−5.**08**, 2.60)
anti-VZV	695	99.7	247	100.0	−0.29 (−1.**22**, 1.71)

N, number of participants with both pre- and post-vaccination results available (except for anti-VZV, which also includes participants without pre-vaccination results available); DT, diphtheria toxoid; FHA, filamentous hemagglutinin; PRN, pertactin; PT, pertussis toxoid; TT, tetanus toxoid, VZV, varicella zoster virus.

BRR, booster response rate (for anti-DT, -FHA, -PRN, -PT, and -TT antibodies): percentage of participants with a booster response for DT, TT, FHA, PRN, or PT antigens (see definitions of booster response in Patients and Methods).

SRR, seroresponse rate (for anti-VZV only): percentage of participants with antibody concentration above the seroresponse threshold (≥75 mIU/mL).

^a^Standardized asymptotic 97.5% confidence interval.

Bold values indicate non-inferiority criterion met. Non-inferiority criteria: lower limit of the two-sided 97.5% CI for the group difference in BRRs or SRRs at D42 (MMR-RIT minus MMR II) ≥-10% for DT, TT, PT, FHA, and PRN antigens and ≥-5% for anti-VZV antibodies.

**Table 5.** Geometric mean antibody concentrations or titers to the co-administered vaccines at Day 42 (according-to-protocol cohort for immunogenicity, sub-cohort 1).

	MMR-RIT		MMR II		Adjusted GMC/GMT ratio (MMR-RIT over MMR II) % (97.5% CI)^a^
	N	Adjusted GMC/GMT	N	Adjusted GMC/GMT	
anti-PV 1 (ED_50_)	669	1636.5	238	1558.4	1.05 (**0.88**, 1.25)
anti-PV 2 (ED_50_)	653	2032.7	233	2197.3	0.93 (**0.78**, 1.09)
anti-PV 3 (ED_50_)	590	2794.4	214	2978.8	0.94 (**0.77**, 1.14)
anti-FHA (IU/mL)	684	313.7	243	323.3	0.97 (**0.88**, 1.07)
anti-PRN (IU/mL)	682	399.9	243	417.6	0.96 (**0.84**, 1.09)
anti-PT (IU/mL)	684	76.1	243	73.0	1.04 (**0.92**, 1.18)
anti-VZV (mIU/mL)	695	879.7	247	830.1	1.06 (**0.95**, 1.18)

N, number of participants with available results; FHA, filamentous hemagglutinin; PRN, pertactin; PT, pertussis toxoid; PV, poliovirus; VZV, varicella zoster virus.

Adjusted GMC/GMT, geometric mean antibody concentration/titer adjusted for pre-vaccination concentration.

^a^97.5% confidence interval calculated with an ANCOVA model.

Bold values indicate non-inferiority criterion met. Non-inferiority criterion: lower limit of the two-sided 97.5% CI for the adjusted GMC or GMT ratio at D42 (MMR-RIT over MMR II) ≥0.67.

The responses to DTaP-IPV co-administered with MMR-RIT were non-inferior to co-administration with MMR II in terms of BRRs to diphtheria, tetanus, and pertussis antigens at D42, as the lower limit of the 97.5% CIs of the difference in BRR (MMR-RIT minus MMR II) was ≥-10% (predefined threshold) for anti-diphtheria toxoid (DT), anti-tetanus toxoid (TT), anti-pertussis toxoid (PT), anti-filamentous hemagglutinin (FHA), and anti-pertactin (PRN) antibodies (Table 4).

Non-inferiority of DTaP-IPV co-administered with MMR-RIT compared to co-administration with MMR II in terms of antibody concentrations or titers against polio and pertussis antigens was demonstrated, as the lower limit of the 97.5% CIs of the adjusted GMC/GMT ratios (MMR-RIT over MMR II) was ≥0.67 for anti-PT, anti-FHA, anti-PRN antibodies and for antibodies against the 3 poliovirus (PV) strains (Table 5).

Non-inferiority of MMR-RIT compared to MMR II in terms of SRR to the VV component was proven, as the lower limit of the 97.5% CIs of the difference in SRR (MMR-RIT minus MMR II) at D42 was above the predefined threshold of −5% for anti-VZV antibody (Table 4). Non-inferiority of VV in terms of antibody concentration against VZV was also met, as the lower limit of the 97.5% CIs of the adjusted GMC ratio (MMR-RIT over MMR II) was ≥0.67 (Table 5).

Overall, we demonstrated non-inferiority of MMR-RIT to MMR II in terms of immune responses to measles, mumps, and rubella viruses, and absence of clinically relevant impact on the immune responses to the antigens contained in the co-administered DTaP-IPV and VV.

### Reactogenicity and safety

A third sub-cohort was included in the study (sub-cohort 3) to assess the reactogenicity and safety of MMR-RIT and MMR II when administered alone. Safety events were also recorded in sub-cohorts 1 and 2. Reactogenicity and safety were assessed in each sub-cohort separately.

**Figure 3.**
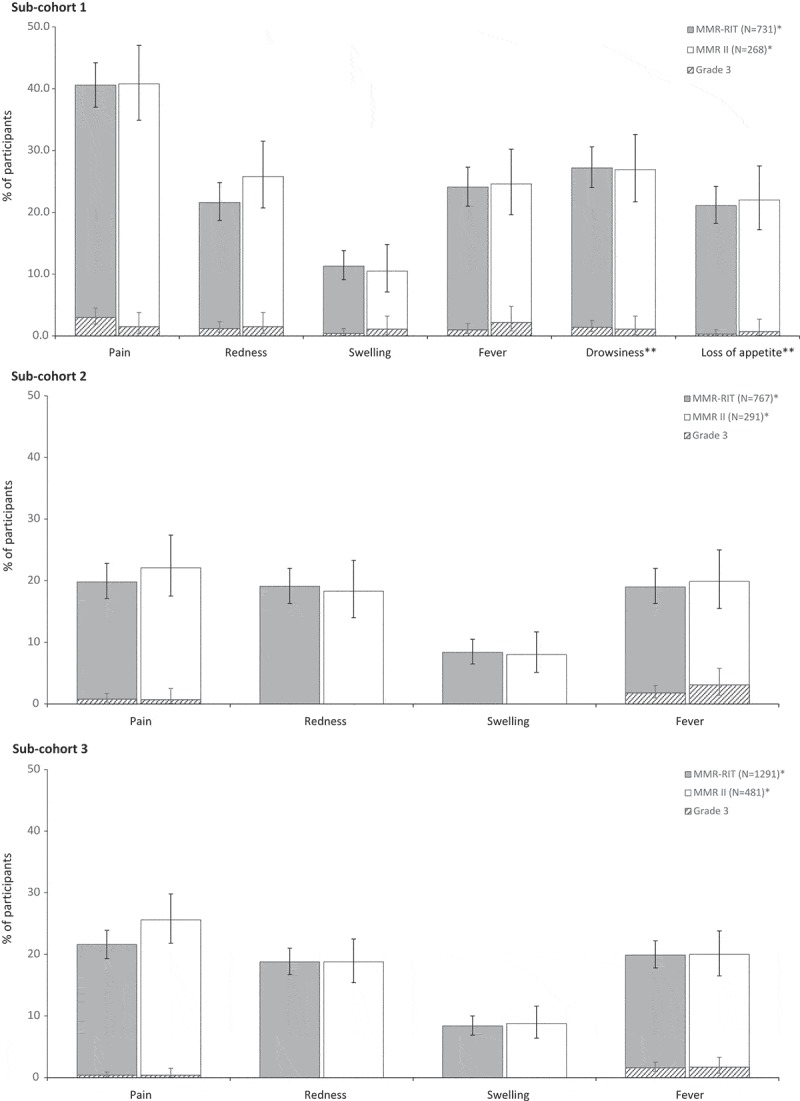
Incidence, in each sub-cohort, of solicited injection site adverse events (Day 0–3), fever (Day 0–42), and drowsiness and loss of appetite (Day 0–3; only assessed in sub-cohort 1) (total vaccinated cohort). Footnote: N, number of participants with at least 1 vaccine administration documented.*Except for fever, drowsiness, and loss of appetite in sub-cohort 1 (MMR-RIT, N = 731 and MMR II, N = 268); fever in sub-cohort 2 (MMR-RIT, N = 767 and MMR II, N = 291); and fever in sub-cohort 3 (MMR-RIT, N = 1291 and MMR II, N = 481). Children in sub-cohort 1 received either MMR-RIT or MMR II together with DTaP-IPV and VV; children in sub-cohorts 2 and 3 received either MMR-RIT or MMR II alone.The injection site adverse events (i.e., pain, redness, and swelling) refer to the site of MMR vaccine injection. Fever: temperature ≥38.0°C. Grade 3 was defined as: limb spontaneously painful or child cried when limb was moved (pain); diameter >50 mm (redness, swelling); temperature >39.5°C (fever); adverse event preventing normal, everyday activities (drowsiness); not eating at all (loss of appetite). The error bars represent the upper and lower limits of the exact two-sided 95% confidence intervals.

In each sub-cohort, the reactogenicity profile was similar between MMR-RIT and MMR II groups (Figure 3). The most common solicited local AE in all 3 sub-cohorts was pain at the MMR injection site. More children reported pain in sub-cohort 1 (40.6% in MMR-RIT; 40.8% in MMR II) than in sub-cohort 2 (19.8% in MMR-RIT; 22.1% in MMR II) and sub-cohort 3 (21.6% in MMR-RIT; 25.6% in MMR II) (Figure 3).

The most common solicited general AE between D0–42 was fever ≥38.0°C (19.0%–24.1% in MMR-RIT; 19.9%–24.6% in MMR II) (Figure 3), followed by rash of any type (4.3%–8.3% in MMR-RIT; 4.1%–10.4% in MMR II) (Table 6).

**Table 6.** Incidence of rash, parotid/salivary gland swelling, and febrile convulsions/headaches (Day 0–42) (total vaccinated cohort).

	Sub-cohort 1		Sub-cohort 2		Sub-cohort 3	
n (%)	MMR-RIT (N = 731)	MMR II (N = 268)	MMR-RIT (N = 767)	MMR II (N = 291)	MMR-RIT (N = 1291)	MMR II (N = 481)
Rash						
Any rash	61 (8.3)	28 (10.4)	37 (4.8)	12 (4.1)	56 (4.3)	23 (4.8)
Grade 3	3 (0.4)	0 (0.0)	1 (0.1)	0 (0.0)	3 (0.2)	0 (0.0)
Measles/rubella-like	14 (1.9)	5 (1.9)	3 (0.4)	2 (0.7)	4 (0.3)	2 (0.4)
Varicella-like*	4 (0.5)	3 (1.1)	-	-	-	-
Parotid/salivary gland swelling	0 (0.0)	0 (0.0)	0 (0.0)	1 (0.3)	1 (0.1)	1 (0.2)
Grade 3	0 (0.0)	0 (0.0)	0 (0.0)	0 (0.0)	0 (0.0)	0 (0.0)
Febrile convulsion/headaches	0 (0.0)	2 (0.7)	1 (0.1)	0 (0.0)	0 (0.0)	0 (0.0)
Grade 3	0 (0.0)	0 (0.0)	0 (0.0)	0 (0.0)	0 (0.0)	0 (0.0)

N, number of participants with the administered dose; n (%), number (percentage) of participants reporting the specified symptom.

Grade 3 was defined as: adverse event preventing normal, everyday activities (any rash, febrile convulsion/headaches); swelling with accompanying general symptoms (parotid/salivary gland swelling).

* Varicella-like rash was assessed only in sub-cohort 1 as a solicited adverse event of interest after varicella vaccine administration.

We also assessed fever between D5–12. The incidence was 4.8% in MMR-RIT and 3.4% in MMR II (sub-cohort 1), 4.3% in MMR-RIT and 2.7% in MMR II (sub-cohort 2), and 5.9% in MMR-RIT and 5.0% in MMR II (sub-cohort 3). Grade 3 fever (>39.5°C) between D5–12 was reported in ≤1.4% of children across vaccine groups and sub-cohorts.

Measles/rubella-like rash was reported in ≤1.9% of children across vaccine groups and sub-cohorts (Table 6). Varicella-like rash, assessed only in sub-cohort 1 as a solicited symptom of interest after VV vaccination, was reported in 4 children of the MMR-RIT group and 3 of the MMR II group. Neurological signs or symptoms suggestive of meningeal irritation were reported in 3 children: 1 case of febrile convulsion in the MMR-RIT group and 2 cases of headache in the MMR II group (Table 6). Parotid gland swelling was reported in 1 child in the MMR-RIT group and 2 children in the MMR II group (Table 6).

We also assessed drowsiness and loss of appetite in sub-cohort 1, as solicited symptoms of interest after DTaP-IPV vaccination. Up to 27.2% of children reported drowsiness and up to 22.0% reported loss of appetite (Figure 3), with a similar incidence between the MMR-RIT and MMR II groups.

In each sub-cohort, the incidence of unsolicited AEs, serious AEs (SAEs) and new onset chronic diseases (NOCDs) were in comparable ranges between the MMR-RIT and MMR II groups (Table 7). The most common unsolicited AEs were cough in sub-cohort 1, and nasopharyngitis in sub-cohorts 2 and 3. Unsolicited AEs of grade 3 severity were reported in ≤3.7% of children across vaccine groups and sub-cohorts (Table 7). The incidence of SAEs in this study was ≤1.9% (Table 7); 1 SAE (generalized rash) in a child in the MMR-RIT group was considered to be causally related to the vaccination. All children reporting SAEs recovered and all SAEs were resolved before the study end. The incidence of NOCDs across treatment groups and sub-cohorts was ≤1.3%; most of them were newly diagnosed allergies. The most common NOCDs were allergic rhinitis in sub-cohorts 1 and 3, and eczema in sub-cohort 2. There were no AEs leading to premature discontinuation of study vaccine or withdrawal from the study. No fatal events were reported.

**Table 7.** Incidence of reported unsolicited adverse events (Day 0–42), serious adverse events, and NOCDs (Day 0–180) (total vaccinated cohort).

	Sub-cohort 1		Sub-cohort 2		Sub-cohort 3	
n (%)	MMR-RIT (N = 802)	MMR II (N = 298)	MMR-RIT (N = 796)	MMR II (N = 303)	MMR-RIT (N = 1319)	MMR II (N = 489)
Unsolicited AEs (≥1 symptom)	276 (34.4)	90 (30.2)	314 (39.4)	112 (37.0)	508 (38.5)	186 (38.0)
Grade 3^a^	24 (3.0)	11 (3.7)	19 (2.4)	10 (3.3)	29 (2.2)	11 (2.2)
SAEs (any, ≥1 SAE)	4 (0.5)	0 (0.0)	14 (1.8)	1 (0.3)	25 (1.9)	9 (1.8)
NOCDs (any, ≥1 NOCD)	8 (1.0)	4 (1.3)	6 (0.8)	0 (0.0)	11(0.8)	3 (0.6)

AE, adverse event; SAE, serious adverse event; N, number of participants with the administered dose; n (%), number (percentage) of participants reporting an AE at least once; NOCDs, new onset chronic diseases.

^a^Unsolicited AEs of grade 3 intensity were those preventing normal, everyday activities.

## Discussion

To our best knowledge, this study is the first comparing MMR-RIT with MMR II when both were administrated as a second dose in children 4–6 years of age with or without other vaccines routinely given at the same timing as per recommended schedule in many countries. We showed that the immunogenicity of the MMR-RIT vaccine, in terms of SRRs and antibody GMCs, was non-inferior to that of the USA standard of care, MMR II, when given as a second dose to children 4–6 years of age with or without co-administration of DTaP-IPV and VV. The immune responses to the co-administered vaccines were also not affected by which MMR vaccine (either MMR-RIT or MMR II) was administered. The safety profile of MMR-RIT was comparable to that of MMR II when administered with or without DTaP-IPV and VV, and we did not observe any new safety concerns or adverse events reported at higher rates than expected.

Children in sub-cohort 1 of the current study received DTaP-IPV and VV along with either MMR-RIT or MMR II. One previous study by Klein and colleagues assessed the DTaP-IPV immune responses in children who were co-administered DTaP-IPV, MMR II, and VV (same doses of the recommended immunization schedule as in the present study).^13^ They found BRRs of ≥95% for antibodies to the DTaP-IPV components. The BRRs observed in sub-cohort 1 of our study are in line with those results (of note, we used definitions of booster response that are similar or more stringent than those used by Klein and colleagues).

In a different, previous study conducted in Australia, the immunogenicity and safety of a second dose of MMR-RIT was assessed when co-administered with DTaP-IPV – but not VV – in children 4–6 years old.^14^ In that study by Marshall and colleagues, all children in the DTaP-IPV + MMR-RIT group showed seroresponse for antibodies against measles, mumps, and rubella 1 month after vaccination, in line with the SRRs of 99.9%–100% observed in sub-cohort 1 of our study.^14^

In a multi-country study by Gillet and colleagues, a second dose of MMR-RIT co-administered with VV in children 2–6 years old elicited robust immune responses, with ≥99% of seropositive children after vaccination.^15^ Sub-cohort 1 of our study showed similar SRRs, even though we used more stringent seroresponse thresholds than those used by Gillet et al.

The immunogenicity of a second dose of MMR-RIT and MMR II administered alone has also been studied. In a previous study conducted in Korea, Lee and colleagues showed 100% seroconversion and similar antibody GMTs after a second dose of either MMR vaccine was administered to children 4–6 years old.^16^ The immunogenicity results of sub-cohort 2 of our study are in line with those by Lee and colleagues, even though our seroresponse thresholds were more stringent than those used by Lee et al.

In our study, children receiving either MMR-RIT or MMR II had comparable safety profiles. Solicited general AEs in our study were reported at similar rates as in children who were co-administered DTaP-IPV and MMR vaccines in 2 previous studies,^14,17^ and also in children who were co-administered DTaP-IPV, MMR II, and VV in another study.^13^ The incidence of solicited local AEs in our study is in line with that reported in the previous study conducted in Australia.^14^ However, solicited local AEs in our study were less frequent than in the study by Klein and colleagues.^13^

Children who received the co-administered DTaP-IPV and VV in our study, regardless of the MMR vaccine they received, showed a higher incidence of pain at the MMR injection site compared to children who did not receive the co-administered vaccines. A previous study where DTaP-IPV was co-administered with MMR II reported a high incidence of pain (60.2%) at the DTaP-IPV injection site; MMR injection site symptoms were not assessed in that study.^17^ In our study, the perception of pain at the MMR injection site in children who also received DTaP-IPV and VV may have been affected by the higher pain at the DTaP-IPV injection site, even though the vaccines were administered at different sites. Pain at one site may have increased the attention (and reporting) of pain at other sites by the parents/legally acceptable representatives (LARs), and DTaP-IPV may also have caused generalized muscular pain.

In the case of mumps vaccine, many attenuated strains have been used widely including Jeryl Lynn, Leningrad, Urabe, Zagreb and Rubini.^18^ Several vaccines derived from the Urabe AM9 mumps strain were withdrawn from the market due to an excessive number of vaccine-associated aseptic meningitis.^19^ A similar association with aseptic meningitis has also been a matter of concern for other mumps vaccine strains such as Leningrad and Zagreb.^20^ The mumps virus strain of MMR-RIT is RIT 4385, derived from the Jeryl Lynn B strain used in the MMR II vaccine. Based on available safety and efficacy evidence, the Jeryl Lynn strain was proved to display the most favourable benefit-risk profile.^21^ However, since aseptic meningitis may be a common event among recipients of mumps vaccine, we recorded any signs of neurological symptoms suggestive of meningeal irritation as safety outcome. The results of the present study add further evidence to long-term follow-up studies with Jeryl Lynn-containing MMR vaccine confirming the general safety profile of MMR-RIT and MMR II vaccines. Although a number of mumps cases have occurred in vaccinated individuals during recent mumps outbreaks, no other mumps vaccine strain is available at present with equivalent or better effectiveness and similar safety profile than the currently used Jeryl Lynn strain.^20,21^

Taking into account the randomization ratio of 3:1 in each sub-cohort, the rates of SAEs and NOCDs suggest an incidence of such events being evenly spread among vaccine groups. For each serious adverse event, information regarding day of onset, duration, intensity, causality and outcome was collected. Importantly, only one case SAE in the MMR-RIT group was considered related to vaccination by the investigator and all subjects recovered with all SAEs resolved before study end. The present trial did not assess AEs as a primary objective (i.e., a statistically powered objective) but only as secondary objective (i.e., non-statistically powered objective); other studies in our clinical trial program are evaluating safety outcomes (fever as solicited AE) as primary (and powered) objective.^22^ As a result, in this study the safety and reactogenicity assessments were only descriptive. The record of rare events with a very low incidence such as SAEs and NOCDs would require a large sample size to detect enough cases. In this regard, phase III studies do not allow assessing any statistical differences with respect to the incidence of SAEs and NOCDs. Large post-marketing studies and good-quality safety databases, such as the ones used to assess the safety of MMR II and MMR-RIT, are a more convenient method to detect events with very low risk.

This study has some limitations. The rationale for administering MMR-RIT along with DTaP-IPV and VV in this study was to reflect the clinical practice of childhood routine immunization programs. The study design did not include groups of children who received each of the co-administered vaccines alone or in pairs with either MMR-RIT or MMR II – a design that could have helped us better understand the relative contribution of each vaccine to the effects observed (e.g., higher pain at the MMR injection site observed in sub-cohort 1). However, it is recommended not to withhold or delay the administration of vaccines routinely used in children, as it is critical to achieve protection against all antigens in a timeframe as short (and adequate) as possible.^11^

In this study, we aimed at comparing MMR-RIT with the current standard of care in the USA (MMR II). Consequently, no placebo group was included and only the safety profile relative to a similar vaccine was assessed.

Most of the children enrolled in this study were from the USA. This limited geographical focus was based on the need to obtain data for the target population, as this study was intended to support licensure of MMR-RIT in the USA. However, we also included children from South Korea and Taiwan, which increases the generalizability of the study results.

While immunogenicity of the measles and rubella components has been universally accepted, the evaluation for the mumps component has been a complicated matter, due to different sensitivities and accuracies of the assays.^18^ In this regard, a potential limitation of this study was the use of an ELISA assay to assess mumps immunogenicity, instead of using a functional assay such as plaque-reduction neutralization assays, which could distinguish neutralizing from non-neutralizing antibodies. We used ELISA since it is the most widely accepted assay for assessing mumps immunogenicity and it has been used in numerous previous studies of MMR-containing vaccines. Another consideration relates to the fact that there is no serological correlate of protection for mumps.^23–25^ The implications of immunogenicity data for mumps should hence be considered cautiously and emphasize the need to take into account other variables such as epidemiological data. However, the results showed that the immune response elicited by MMR-RIT to the different MMR components was non-inferior to that elicited by MMR II, which stands for the primary objectives of this clinical trial.

In conclusion, our results showed non-inferiority of MMR-RIT compared with the standard of care in the USA when administered in children 4–6 years of age with or without other vaccines routinely recommended at that age – namely DTaP-IPV and VV – without affecting the immunogenicity of these co-administered vaccines.

## Patients and methods

### Study design and participants

We conducted a phase IIIa, observer-blind, randomized, controlled study (ClinicalTrials.gov: NCT01621802) between June 2012 and November 2015 in 70 sites in 3 countries: South Korea, Taiwan, and the USA.

**Figure 4.**
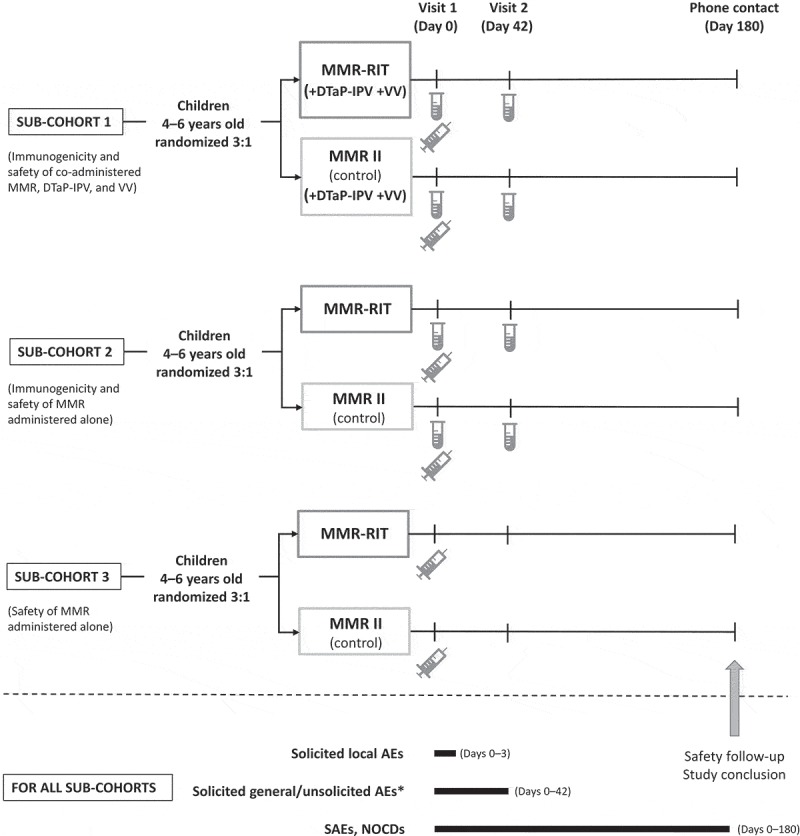
Study design. Footnote: 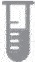, blood sampling; 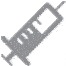, vaccine administration (includes administration of DTaP-IPV and VV in sub-cohort 1); AEs, adverse events; DTaP-IPV, diphtheria, tetanus, acellular pertussis, and inactivated polio vaccine; NOCDs, new onset chronic diseases; SAEs, serious adverse events; VV, varicella vaccine.*Drowsiness and loss of appetite were recorded as solicited general AE from Day 0 to Day 3 in sub-cohort 1 only.

In this study, we enrolled healthy children 4–6 years of age in 3 sub-cohorts based on country and site (Figure 4). Sub-cohort 1 consisted of children enrolled only in the USA; sub-cohorts 2 and 3 consisted of children from all 3 countries. Children in each sub-cohort were then randomized in a 6:1:1 ratio to receive 1 dose of MMR-RIT, MMR II (lot 1), or MMR II (lot 2). As the 2 lot groups of MMR II were pooled for the analyses, the overall randomization ratio in each sub-cohort was 3:1 (MMR-RIT:MMR II). All children in sub-cohort 1 also received DTaP-IPV and VV (1 dose each, which corresponded to the fifth dose of DTaP-containing vaccine, fourth dose of IPV and second dose of VV in the recommended immunization schedule for the participants).

Randomization of the MMR vaccines was performed at GSK (Rixensart, Belgium) using MATEX for Statistical Analysis Systems (SAS), with a blocking scheme of 6:1:1 within each sub-cohort. DTaP-IPV and VV were not randomized. Each site performed the treatment allocation using a central randomization system on internet (SBIR) with sub-cohort stratification and a minimization procedure accounting for site within each sub-cohort.

Due to potential color differences between reconstituted MMR-RIT and MMR II, the health care personnel who prepared and administered the vaccines were not blinded to the treatment group information; these personnel were not involved in the assessment of study endpoints. All data were then collected in an observer-blind manner: the vaccinees and their parents/LARs, the personnel involved in the laboratory tests, and those responsible for evaluating any study endpoint were blinded to the treatment.

This study consisted of 2 site visits (D0 and D42) and 1 phone contact for safety follow-up (D180; Figure 4). Vaccines were administered at D0. Blood samples were collected, from cohorts 1 and 2 only, at D0 and D42.

Children 4–6 years of age in stable health as determined by the investigator were eligible for the study if their parents/LARs were to comply with requirements of the protocol and provided written informed consent, and if children met the following criteria based on assessment of their clinical history: no history of measles, mumps, or rubella diseases, no exposure to measles, mumps, or rubella viruses within 30 days prior to enrollment, and receipt of only 1 previous dose of a combined MMR vaccine or MMR and varicella virus-containing vaccine (MMR II, *M-M-R VaxPro* or P*roQuad (MMRV*), Merck & Co Inc.), administered in their second year of life.

Additional inclusion criteria for children to be enrolled in sub-cohort 1 were 1) previous receipt of 3 DTaP-containing vaccine doses as *Infanrix* (diphtheria and tetanus toxoids and acellular pertussis vaccine, adsorbed, GSK) and/or *Pediarix* (diphtheria and tetanus toxoids and acellular pertussis vaccine, adsorbed, hepatitis B [recombinant], inactivated poliovirus vaccine, GSK), plus a fourth dose as *Infanrix*; and 2) previous receipt of 1 dose of a varicella virus-containing vaccine (*Varivax* or *ProQuad (MMRV*), Merck & Co Inc.) in the second year of life.

Children in care and those who received more than the indicated number of previous doses of MMR vaccine (all sub-cohorts) or DTaP or VV (sub-cohort 1 only) were excluded from the study, as well as those planned to receive any non-study vaccine during the period from 30 days before enrollment to D41 (except live intranasal or inactivated influenza vaccine, allowed at any time). Details on these criteria, as well as additional exclusion criteria, are described in the supplementary material.

Independent ethics review committees or institutional review boards approved the study protocol, a summary of which is available at https://www.gsk-clinicalstudyregister.com/study/115158. The study was conducted in accordance with the Declaration of Helsinki and the Good Clinical Practice guidelines. The parent(s)/LAR(s) of each participant provided written informed consent before enrollment.

External organizations were contracted to help conduct the research (management and monitoring of the study, sample management, and laboratory tests).

### Study objectives

This study had 4 co-primary objectives to evaluate the immunogenicity of MMR-RIT compared to MMR II, when administered with and without DTaP-IPV and VV, in terms of SRR and antibody concentrations to the MMR components (non-inferiority objectives; see “Statistical analyses” for details).

Secondary objectives included 1) evaluation of immunogenicity of DTaP-IPV and VV, in terms of BRRs or SRRs, and antibody concentrations to the components of DTaP-IPV and VV, when they were co-administered with MMR-RIT as compared to when co-administered with MMR II (non-inferiority objectives; see “Statistical analyses” for details); and 2) assessment of the reactogenicity and safety of MMR-RIT and MMR II vaccines in each sub-cohort separately.

### Study vaccines

The MMR-RIT vaccine contains live attenuated measles virus (Schwarz strain) ≥10^3.0^ cell culture infectious dose 50 (CCID_50_), mumps virus (RIT4385 strain) ≥10^4.3^ CCID_50_, rubella virus (Wistar RA 27/3 strain) ≥10^3.0^ CCID_50_, anhydrous lactose, sorbitol, mannitol, amino acids, and neomycin.

The MMR II vaccine contains live attenuated measles virus (Moraten Edmonston-Enders strain) ≥10^3.0^ tissue culture infectious dose 50 (TCID_50_), mumps virus (Jeryl Lynn strain) ≥10^4.1^ TCID_50_, rubella virus (Wistar RA 27/3 strain) ≥10^3.0^ TCID_50_, sorbitol, sodium phosphate, sucrose, sodium chloride, hydrolyzed gelatin, recombinant human albumin, fetal bovine serum, and neomycin.

The composition of DTaP-IPV (*Kinrix*, GSK) and VV (VARIVAX, Merck & Co Inc) has been described elsewhere.^26,27^ Each child received either MMR-RIT or MMR II subcutaneously in the right upper arm (triceps region). In sub-cohort 1, VV was administered subcutaneously in the left upper arm (triceps region), and DTaP-IPV intramuscularly in the left deltoid.

### Immunogenicity assessments

Immunogenicity assessments were performed on blood samples taken at D0 and D42 from children in sub-cohorts 1 and 2. Sera were stored and transported at −20°C until assayed at a designated laboratory using standardized and validated procedures. IgG antibodies to measles, rubella, and VZV were measured using a commercial enzyme-linked immunosorbent assay (ELISA) kit, *Enzygnost* (Dade Behring) at NÉOMED-LABS Inc., Quebec, Canada. IgG antibodies against mumps were measured using an ELISA kit at Pharmaceutical Product Development, Inc., Pennsylvania, USA. IgG antibodies against DT, TT, PT, FHA, and PRN were measured using an in-house ELISA (GSK, Belgium), whereas antibodies against PV Sabin types 1, 2, and 3 were measured using an in-house virus micro-neutralization assay (GSK, Belgium).^28^

To define seroresponses and booster responses, we used antibody concentration thresholds that were accepted by the Food and Drug Administration (FDA) as endpoints defining active immunization offering clinical benefit. The anti-VZV threshold was accepted by the FDA as threshold commonly used in previous studies.^29^ Seroresponse was defined as an IgG antibody concentration ≥200 mIU/mL for anti-measles, ≥10 EU/mL for anti-mumps, ≥10 IU/mL for anti-rubella, and ≥75 mIU/mL for anti-VZV, at D42, and did not take into account pre-vaccination concentrations (since most of the participants were expected to be seroresponsive before their second dose of MMR vaccine and VV vaccine, administered in this study). Booster responses for PT, FHA, and PRN antigens were defined as:

- For participants with pre-vaccination antibody concentration below the assay cut-off (i.e., <2.693 IU/mL for anti-PT, <2.046 IU/mL for anti-FHA, and <2.187 IU/mL for anti-PRN): post-vaccination antibody concentration ≥4 times the assay cut-off.
- For participants with pre-vaccination antibody concentration between the assay cut-off and 4 times above the assay cut-off: post-vaccination antibody concentration ≥4 times the pre-vaccination antibody concentration.
- For participants with pre-vaccination antibody concentration ≥4 times the assay cut-off: post-vaccination antibody concentration ≥2 times the pre-vaccination antibody concentration.

Booster responses for DT and TT antigens were defined as:

- For participants with pre-vaccination concentration <0.1 IU/mL (i.e., below the seroprotection threshold): post-vaccination antibody concentrations ≥0.4 IU/mL.
- For participants with pre-vaccination concentration ≥0.1 IU/mL: an increase in antibody concentrations ≥4 times the pre-vaccination concentration 43 days after vaccination.

### Reactogenicity and safety assessments

In all the sub-cohorts, solicited local AEs (injection site pain, redness, and swelling) were recorded from D0 to D3. MMR-specific solicited general AEs were recorded from D0 to D42 (Figure 4) and included: fever (defined as temperature ≥38.0°C), rash (including measles/rubella-like and any rash), swelling of the parotid or other salivary glands, symptoms suggestive of meningeal irritation including febrile convulsions and headaches. In sub-cohort 1, drowsiness and loss of appetite were also recorded from D0 to D3, and varicella-like rash was recorded from D0 to D42, as solicited general AEs (Figure 4).

Unsolicited AEs were documented from D0 to D42, whereas SAEs were documented throughout the entire study period (D0–180). NOCDs (e.g., autoimmune disorders, asthma, type I diabetes, vasculitis, celiac disease, conditions associated with sub-acute or chronic thrombocytopenia, and allergies) were recorded from D0 to D180.

We graded solicited AEs according to their intensity (grade 1–3). Grade 3 was defined as: limb spontaneously painful or child cried when limb was moved (pain); redness or swelling of diameter >50 mm; temperature >39.5°C (fever); AE preventing normal, everyday activities (any rash, febrile convulsion, drowsiness, unsolicited AEs); swelling with accompanying general symptoms (parotid/salivary gland swelling); not eating at all (loss of appetite). All solicited local (injection site) reactions were considered causally related to vaccination. Causality of all other AEs was assessed by the investigator.

### Statistical analyses

We planned to enroll 4000 children in this study: 1096 in sub-cohort 1 (MMR-RIT, N = 822; MMR II, N = 274), 1096 in sub-cohort 2 (MMR-RIT, N = 822; MMR II, N = 274), and 1808 in sub-cohort 3 (MMR-RIT, N = 1356; MMR II, N = 452). Assuming a 20% non-evaluable rate in the according-to-protocol cohort for immunogenicity, 876 children (MMR-RIT, N = 657; MMR II, N = 219) would be evaluable in each of the sub-cohorts 1 and 2.

All the immunogenicity objectives (primary and secondary) were statistically powered. The 4 co-primary objectives were assessed in parallel. To control the type I error below 2.5%, a Bonferroni adjustment was used to compare MMR-RIT and MMR II independently in either sub-cohort 1 or 2, with a 1.25% nominal type I error for the group comparison in each sub-cohort. In addition, a hierarchical procedure was used for the secondary objectives. The global power to reach all non-inferiority objectives for both sub-cohorts 1 and 2 was 93% assuming independence of sub-cohorts.

All the safety and reactogenicity analyses were descriptive only, and were conducted on the total vaccinated cohort, which included all vaccinated subjects with at least 1 vaccine administration documented. We tabulated, for each sub-cohort separately, the number and percentage of children reporting each of the safety and reactogenicity variables assessed.

All the immunogenicity analyses were conducted on the according-to-protocol cohort for immunogenicity, which included participants who received the vaccine(s) as per protocol, met eligibility criteria, complied with protocol-defined procedures, and had post-vaccination (D42) immunogenicity results for at least 1 of the 3 MMR vaccine components as appropriate for the sub-cohort. For the prespecified immunogenicity analyses, and for sub-cohorts 1 and 2 separately, the antibody concentrations or titers were summarized by antibody GMCs or GMTs with their 95% CIs. SRRs for antibodies to measles, mumps, rubella, and varicella (defined as percentage of children showing seroresponse to these antibodies), and BRRs for antibodies to DT, TT, and PT (defined as percentage of children showing booster response to these antibodies) were tabulated with their exact 95% CIs. We also tabulated the difference in SRR or BRR between groups (MMR-RIT minus MMR II) with its asymptotic standardized 97.5% CI, and the adjusted GMC or GMT ratio between groups (MMR-RIT over MMR II) with its 97.5% CI, for each prespecified antigen at D42.

#### Primary non-inferiority objectives and criteria

- To demonstrate the non-inferiority of MMR-RIT vaccine compared to MMR II vaccine, when administered with DTaP-IPV and VV (sub-cohort 1), in terms of SRRs to measles, mumps, and rubella viruses at D42. This was reached if the lower limit of the two-sided 97.5% CI for the group difference in SRRs at D42 (MMR-RIT SRR minus MMR II SRR) was ≥-5% for the 3 antigens.
- To demonstrate the non-inferiority of MMR-RIT vaccine compared to MMR II vaccine, when administered with DTaP-IPV and VV (sub-cohort 1), in terms of antibody concentrations to measles, mumps, and rubella viruses at D42. Non-inferiority was demonstrated if the lower limit of the two-sided 97.5% CI for the adjusted GMC ratio at D42 (MMR-RIT GMC over MMR II GMC) was ≥0.67 for antibodies to the 3 antigens.
- To demonstrate the non-inferiority of MMR-RIT vaccine compared to MMR II vaccine, when administered without DTaP-IPV and VV (sub-cohort 2), in terms of SRRs to measles, mumps, and rubella viruses at D42. This was reached if the lower limit of the two-sided 97.5% CI for the group difference in SRRs at D42 (MMR-RIT SRR minus MMR II SRR) was ≥-5% for the 3 antigens.
- To demonstrate the non-inferiority of MMR-RIT vaccine compared to MMR II vaccine, when administered without DTaP-IPV and VV (sub-cohort 2), in terms of antibody concentrations to measles, mumps, and rubella viruses at D42. Non-inferiority was demonstrated if the lower limit of the two-sided 97.5% CI for the adjusted GMC ratio at D42 (MMR-RIT GMC over MMR II GMC) was ≥0.67 for antibodies to the 3 antigens.

#### Secondary non-inferiority objectives and criteria

- To demonstrate the non-inferiority in terms of SRRs and antibody concentrations to VZV at D42 when VV is administered with MMR-RIT and DTaP-IPV as compared to when VV is administered with MMR II and DTaP-IPV (sub-cohort 1). This was proven if 1) the lower limit of the two-sided 97.5% CI for the group difference in SRRs at D42 (MMR-RIT SRR minus MMR II SRR) was ≥-5% for anti-VZV antibody, and 2) the lower limit of the two-sided 97.5% CI for the adjusted GMC ratio at D42 (MMR-RIT GMC over MMR II GMC) was ≥0.67 for anti-VZV antibody.
- To demonstrate the non-inferiority in terms of BRRs to DT, TT, PT, FHA, and PRN when DTaP-IPV is administered with MMR-RIT and VV as compared to when DTaP-IPV is administered with MMR II and VV (sub-cohort 1). This was reached if the lower limit of the two-sided 97.5% CI for the group difference in BRR at D42 (MMR-RIT BRR minus MMR II BRR) was ≥-10% for DT, TT, PT, FHA, and PRN antigens.
- To demonstrate the non-inferiority in terms of antibody titers to PV types 1, 2, and 3 at D42 when DTaP-IPV is administered with MMR-RIT and VV as compared to when DTaP-IPV is administered with MMR II and VV (sub-cohort 1). This was demonstrated if the lower limit of the two-sided 97.5% CI for the GMT ratio at D42 (MMR-RIT GMT over MMR II GMT) was ≥0.67 for the 3 PV antigens.
- To demonstrate the non-inferiority in terms of anti-PT, anti-FHA, and anti-PRN antibody concentrations at D42 when DTaP-IPV is administered with MMR-RIT and VV as compared to when DTaP-IPV is administered with MMR II and VV (sub-cohort 1). This was proven if the lower limit of the two-sided 97.5% CI for the adjusted GMC ratio at D42 (MMR-RIT GMC over MMR II GMC) was ≥0.67 for each antibody.

Statistical analyses were performed using the SAS version 9.3 on SAS Drug Development 4.3. The definition of non-inferiority thresholds is provided in the supplemental online material.

### Changes in the conduct of the study

This study was conducted according-to-protocol, and all analyses were performed as planned in the protocol and the statistical analysis plan except for the following: 1) During the course of the study, the ELISA assays used to measure anti-DT, anti-TT, anti-PT, anti-FHA, and anti-PRN IgG antibody concentrations were re-developed and re-validated. The new ELISA was calibrated against the WHO International Standard (NIBSC 06/140) for PT, FHA, and PRN antigens.^30^ 2) The booster response criteria for anti-DT and anti-TT were corrected during the conduct of the study. New statistical outputs were generated, and the BRRs reported in this manuscript are those calculated with the correct criteria.

## Trademark statement

*Priorix, Kinrix, Infanrix*, and *Pediarix* are trademarks owned by or licensed to the GSK group of companies. *M-M-R II, M-M-R VaxPro, ProQuad*, and *VARIVAX* are trademarks of Merck and Co., Inc. *Enzygnost* is a trademark of Dade Behring Marburg GmbH.

## References

[1] McLean HQ, Fiebelkorn AP, Temte JL, Wallace GS. Prevention of measles, rubella, congenital rubella syndrome, and mumps, 2013: summary recommendations of the Advisory Committee on Immunization Practices (ACIP). MMWR Recomm Rep. 2013;62(RR–04):1–34. 23760231

[2] World Health Organization Mumps vaccines: WHO position paper. Wkly Epidemiol Rec. 2007;82(7):49–60.

[3] World Health Organization Rubella vaccines: WHO position paper. Wkly Epidemiol Rec. 2011;86(29):301–316. 21766537

[4] World Health Organization Measles vaccines: WHO position paper. Wkly Epidemiol Rec. 2017;92(17):205–228.28459148

[5] Centers for Disease Control and Prevention Measles–United States, 2000. MMWR Morb Mortal Wkly Rep. 2002;51(6):120–123. 11898926

[6] Centers for Disease Control and Prevention Chapter 20: Rubella In: Epidemiology and prevention of vaccine-preventable diseases. 13th ed. “The Pink Book”: Centers for Disease Control and Prevention, Washington DC; 2012 p. 325–340.

[7] Centers for Disease Control and Prevention Mumps cases and outbreaks. Centers for disease control and prevention; 2018 3 6 [accessed 2018 Mar 13]. https://www.cdc.gov/mumps/outbreaks.html.

[8] Centers for Disease Control and Prevention Measles cases and outbreaks. Centers for disease control and prevention; 2017 10 13 [accessed 2017 Oct 13]. https://www.cdc.gov/measles/cases-outbreaks.html.

[9] European Medicines Agency Priorix. Annex III: Summary of product characteristics, labelling and package leaflet; 2012 [accessed 2018 1 15]. http://www.ema.europa.eu/docs/en_GB/document_library/Referrals_document/Priorix_30/WC500124199.pdf.

[10] Wellington K, Goa KL Measles, mumps, rubella vaccine (Priorix; GSK-MMR): a review of its use in the prevention of measles, mumps and rubella. Drugs. 2003;63(19):2107–2126. doi:10.2165/00003495-200363190-00012. 12962524

[11] European Medicines Agency Guideline on clinical evaluation of new vaccines. EMEA/CHMP/VWP/164653/2005; 2006 http://wwwemaeuropaeu/docs/en_GB/document_library/Scientific_guideline/2009/09/WC500003870pdf.

[12] World Health Organization Guidelines on clinical evaluation of vaccines: regulatory expectations. WHO/DRAFT; 2016 1 27 http://wwwwhoint/biologicals/Clinical_guidelines_27_January_2016pdf.

[13] Klein NP, Weston WM, Kuriyakose S, Kolhe D, Howe B, Friedland LR, Van Der Meeren O An open-label, randomized, multi-center study of the immunogenicity and safety of DTaP-IPV (Kinrix) co-administered with MMR vaccine with or without varicella vaccine in healthy pre-school age children. Vaccine. 2012;30(3):668–674. doi:10.1016/j.vaccine.2011.10.065. 22064267

[14] Marshall H, Nolan T, Roberton D, Richmond P, Lambert S, Jacquet JM, Schuerman L A comparison of booster immunisation with a combination DTPa-IPV vaccine or DTPa plus IPV in separate injections when co-administered with MMR, at age 4-6 years. Vaccine. 2006;24(35–36):6120–6128. doi:10.1016/j.vaccine.2006.05.017. 16822597

[15] Gillet Y, Steri GC, Behre U, Arsene JP, Lanse X, Helm K, Esposito S, Meister N, Desole MG, Douha M, et al Immunogenicity and safety of measles-mumps-rubella-varicella (MMRV) vaccine followed by one dose of varicella vaccine in children aged 15 months-2 years or 2-6 years primed with measles-mumps-rubella (MMR) vaccine. Vaccine. 2009;27(3):446–453. doi:10.1016/j.vaccine.2008.10.064. 19007835

[16] Lee H, Kim HW, Cho HK, Park EA, Choi KM, Kim KH Reappraisal of MMR vaccines currently used in Korea. Pediatr Int. 2011;53(3):374–380. doi:10.1111/j.1442-200X.2010.03244.x. 20831649

[17] Black S, Friedland LR, Schuind A, Howe B, for the GlaxoSmithKline DTaP–IPV Vaccine Study Group Immunogenicity and safety of a combined DTaP-IPV vaccine compared with separate DTaP and IPV vaccines when administered as pre-school booster doses with a second dose of MMR vaccine to healthy children aged 4-6 years. Vaccine. 2006;24(35–36):6163–6171. doi:10.1016/j.vaccine.2006.04.001. 16759769

[18] Santos EM, Silva E Sa GR, Siqueira MM, Martins RM, Camacho LA, von Doellinger Vdos R, Maia ML Immune response to the mumps component of the MMR vaccine in the routine of immunisation services in the Brazilian national immunisation program. Mem Inst Oswaldo Cruz. 2014;109(3):335–339. 24821058 10.1590/0074-0276130351 PMC4131786

[19] Amexis G, Fineschi N, Chumakov K Correlation of genetic variability with safety of mumps vaccine Urabe AM9 strain. Virology. 2001;287(1):234–241. doi:10.1006/viro.2001.1009. 11504558

[20] Kaaijk P, van der Zeijst B, Boog M, Hoitink C Increased mumps incidence in the Netherlands: review on the possible role of vaccine strain and genotype. Euro Surveill. 2008;13:26. 18761918

[21] Peltola H, Kulkarni PS, Kapre SV, Paunio M, Jadhav SS, Dhere RM Mumps outbreaks in Canada and the United States: time for new thinking on mumps vaccines. Clin Infect Dis. 2007;45(4):459–466. doi:10.1086/520028. 17638194

[22] The MMR-162 Study Group Safety and immunogenicity of an upper-range release titer measles-mumps-rubella vaccine in children vaccinated at 12 to 15 months of age: a phase III, randomized study. Hum Vaccin Immunother. 2018:1–11. doi:10.1080/21645515.2018.1502527. PMC6343620 30118386

[23] Chen X, Bailleux F, Desai K, Qin L, Dunning AJ A threshold method for immunological correlates of protection. BMC Med Res Methodol. 2013;13:29. doi:10.1186/1471-2288-13-29. 23448322 PMC3639076

[24] Plotkin SA Correlates of protection induced by vaccination. Clin Vaccine Immunol. 2010;17(7):1055–1065. doi:10.1128/cvi.00131-10. 20463105 PMC2897268

[25] World Health Organization Correlates of vaccine-induced protection: methods and implications. Immunization Vaccines Biol. WHO/IVB/1301; 2013 http://appswhoint/iris/bitstream/handle/10665/84288/WHO_IVB_1301_engpdf;jsessionid=18887B1B09124C870BF9B348E23F93A1?sequence=1.

[26] Food and Drug Administration Kinrix. Package insert; 2018 2 12 https://www.fda.gov/downloads/BiologicsBloodVaccines/Vaccines/ApprovedProducts/UCM241453.pdf.

[27] Food and Drug Administration Varixax. Package insert; 2018 2 12 https://www.fda.gov/downloads/BiologicsBloodVaccines/Vaccines/ApprovedProducts/UCM142813.pdf.

[28] World Health Organization Standard procedure for determining immunity to poliovirus using the microneutralization test. WHO/EPI/GEN/939; 1993 http://appswhoint/iris/bitstream/handle/10665/70486/WHO_EPI_GEN_939_engpdf?sequence=1&isAllowed=y.

[29] Blatter MM, Klein NP, Shepard JS, Leonardi M, Shapiro S, Schear M, Mufson MA, Martin JM, Varman M, Grogg S, et al Immunogenicity and safety of two tetravalent (measles, mumps, rubella, varicella) vaccines coadministered with hepatitis a and pneumococcal conjugate vaccines to children twelve to fourteen months of age. Pediatr Infect Dis J. 2012;31(8):e133–40. doi:10.1097/INF.0b013e318259fc8a. 22622699

[30] World Health Organization WHO international standard pertussis antiserum (Human) 1st IS, NIBSC code: 06/140; 2013 http://www.nibsc.org/documents/ifu/06-140.pdf.

