# Supplementary material for: A second dose of a measles-mumps-rubella vaccine administered to healthy four-to-six-year-old children: a phase III, observer-blind, randomized, safety and immunogenicity study comparing GSK MMR and MMR II with and without DTaP-IPV and varicella vaccines co-administration
Source: Hum Vaccin Immunother. 2019 Feb 20;15(4):786–99. doi: 10.1080/21645515.2018.1554971 (PMC6605865; doi:10.1080/21645515.2018.1554971)
Supplement: Supplemental Material [file khvi-15-04-1554971-s001.docx]

**Supplementary material**

**Details of exclusion criteria**

Children in care were defined as children who have been placed under the control or protection of an agency, organization, institution or entity by the courts, the government, or a government body, acting in accordance with powers conferred on them by law or regulation; the definition of children in care does not include children who are adopted or have an appointed legal guardian.

Other exclusion criteria were:

- Use of any investigational or non-registered product (drug or vaccine) other than the study vaccine during the period starting 30 days before the day of study vaccination(s) (i.e., 30 days prior to Day[D]0) or planned during the entire study period.
- Chronic administration (defined as 14 or more consecutive days) of immunosuppressants or other immune-modifying drugs during the period starting 180 days prior to D0, or any planned administration of immunosuppressive and immune-modifying drugs during the entire study. For corticosteroids, this would mean prednisone ≥0.5 mg/kg/day or equivalent. Inhaled and topical steroids were allowed.
- Administration of immunoglobulins and/or any blood products during the period starting 180 days before entering the study or planned administration from the date of vaccination through the immunogenicity evaluation at D42.
- Concurrently participating in another clinical study, at any time during the study period, in which the subject was or would be exposed to an investigational or a non-investigational product (pharmaceutical product or device).
- Any confirmed or suspected immunosuppressive or immunodeficient condition, based on medical history and physical examination (no laboratory testing required).
- History of allergic disease or reactions likely to be exacerbated by any component of the study vaccine(s), including systemic hypersensitivity to neomycin or gelatin.
- Blood dyscrasias, leukemia, lymphomas of any type, or other malignant neoplasms affecting the bone marrow or lymphatic systems.
- Acute disease at the time of enrollment. Acute disease was defined as the presence of a moderate or severe illness with or without fever. Fever was defined as temperature ≥38°C measured by any age-appropriate route. All vaccines could be administered to children with a minor illness such as diarrhea, mild upper respiratory infection without fever.
- Active untreated tuberculosis according to the participant’s medical history.
- Any other condition which, in the opinion of the investigator, prevented the child from participating in the study.

For children enrolled in sub-cohort 1 (i.e., receiving co-administered diphtheria, tetanus, acellular pertussis, and inactivated polio vaccine [DTaP-IPV] and varicella vaccine [VV]) additional exclusion criteria were:

- Previous vaccination with a second dose of a varicella-containing vaccine.
- Receipt of any varicella-containing vaccine during the period starting 90 days before the day of study vaccination.
- History of varicella/zoster disease.
- Known exposure to varicella/zoster during the period starting 30 days prior to enrollment.
- History of diphtheria, tetanus, pertussis, or poliomyelitis disease.
- Vaccination against diphtheria, tetanus, pertussis or poliomyelitis administered when the child was ≥2 years of age.
- Occurrence of transient thrombocytopenia or neurological complications following an earlier immunization against diphtheria or tetanus toxoids.
- Occurrence of any of the following events after a previous administration of DTP vaccine:
  - A temperature ≥40.6°C during the period starting 48 hours after vaccination not due to another identifiable cause.
  - A collapse or shock-like state (hypotonic-hypo-responsive episode) during the period starting 48 hours after vaccination.
  - Persistent, inconsolable crying lasting 3 hours or more within 48 hours after vaccination.
  - Seizures with or without fever occurring during the period starting 3 days after vaccination.
  - Encephalopathy of unknown etiology occurring during the period starting within 7 days of vaccination of a previous administration of DTP vaccine.
  - Hypersensitivity reaction to any component of the DTaP-IPV or varicella vaccines (e.g., latex).

**Grade 3 definition**

Grade 3 definition for most of the adverse events (AEs) in the study protocol was defined as an AE which prevented normal, everyday activities (in a young child, such a grade 3 AE would, for example, prevent attendance at school and would cause the parent[s]/legally acceptable representative[s] to seek medical advice). This definition reflects the guidance established by the FDA concerning the grading of severity (intensity ranges), in which grade 3 refers to: severe AEs, medically significant but not immediately life-threatening AEs, hospitalization or prolongation of hospitalization indicated, or disabling AEs (the guidance can be checked in the following link: <http://www.fda.gov/downloads/BiologicsBloodVaccines/GuidanceComplianceRegulatoryInformation/Guidances/Vaccines/ucm091977.pdf>). Regarding solicited local AEs such as redness and swelling, the grade 3 definition reflects a diameter size that is currently used in pediatric clinical trials.[^1^](#_ENREF_1)

**Non-inferiority thresholds**

The non-inferiority thresholds are the maximum acceptable margins, as required by the regulatory agency, for the differences between 2 vaccines that are similar and allow to achieve a balance between clinical relevance for protection (i.e. ensuring the new vaccine does not confer a lower protection), and allowing achievable sample sizes that can be recruited. Such margins, custom selected according to each vaccine or antigen, are commonly used for new vaccine developments when a previous vaccine exists that serves as a benchmark based on immunogenicity parameters: 10% for seroconversion rates with influenza vaccines,[^2^](#_ENREF_2) 2-fold ratio of neutralizing antibody titers against smallpox,[^3^](#_ENREF_3) 10% for response rates against polio.[^4^](#_ENREF_4)

**Bibliography**

1. Klein NP, Habanec T, Kosina P, Shah NR, Kolhe D, Miller JM, Hezareh M, Van der Wielen M. Immunogenicity and safety of the quadrivalent meningococcal ACWY-tetanus toxoid conjugate vaccine (MenACWY-TT) in splenectomized or hyposplenic children and adolescents: Results of a phase III, open, non-randomized study. Vaccine. 2018;36(17):2356-63. doi:10.1016/j.vaccine.2018.02.085

2. Bart S, Cannon K, Herrington D, Mills R, Forleo-Neto E, Lindert K, Abdul Mateen A. Immunogenicity and safety of a cell culture-based quadrivalent influenza vaccine in adults: A Phase III, double-blind, multicenter, randomized, non-inferiority study. Hum Vaccin Immunother. 2016;12(9):2278-88. doi:10.1080/21645515.2016.1182270

3. Jackson LA, Frey SE, El Sahly HM, Mulligan MJ, Winokur PL, Kotloff KL, Campbell JD, Atmar RL, Graham I, Anderson EJ, et al. Safety and immunogenicity of a modified vaccinia Ankara vaccine using three immunization schedules and two modes of delivery: A randomized clinical non-inferiority trial. Vaccine. 2017;35(13):1675-82. doi:10.1016/j.vaccine.2017.02.032

4. Rivera L, Pedersen RS, Pena L, Olsen KJ, Andreasen LV, Kromann I, Nielsen PI, Sorensen C, Dietrich J, Bandyopadhyay AS, et al. Immunogenicity and safety of three aluminium hydroxide adjuvanted vaccines with reduced doses of inactivated polio vaccine (IPV-Al) compared with standard IPV in young infants in the Dominican Republic: a phase 2, non-inferiority, observer-blinded, randomised, and controlled dose investigation trial. Lancet Infect Dis. 2017;17(7):745-53. doi:10.1016/s1473-3099(17)30177-9
